# Determining the Independent Risk Factors and Mortality Rate of Nosocomial Infections in Pediatric Patients

**DOI:** 10.1155/2016/7240864

**Published:** 2016-02-15

**Authors:** Fesih Aktar, Recep Tekin, Ali Güneş, Cevat Ülgen, İlhan Tan, Sabahattin Ertuğrul, Muhammet Köşker, Hasan Balık, Duran Karabel, Ilyas Yolbaş

**Affiliations:** ^1^Department of Pediatric Intensive Care Unit, Dicle University Medical School, 21010 Diyarbakir, Turkey; ^2^Dicle University School of Medicine, Clinical Microbiology and Infectious Diseases, 21010 Diyarbakir, Turkey; ^3^Department of Pediatrics, Dicle University Medical School, 21010 Diyarbakir, Turkey; ^4^Department of Pediatric Infectious Diseases, Diyarbakir Children's Hospital, Diyarbakir, Turkey

## Abstract

The objective of this study was to determine the rate, independent risk factors, and outcomes of healthcare-associated infections in pediatric patients. This study was performed between 2011 and 2014 in pediatric clinic and intensive care unit. 86 patients and 86 control subjects were included in the study. Of 86 patients with nosocomial infections (NIs), there were 100 NIs episodes and 90 culture growths. The median age was 32.0 months. The median duration of hospital stay of the patients was 30.0 days. The most frequent pathogens were Coagulase-negative* Staphylococcus*,* Acinetobacter* spp.,* Klebsiella* spp., and* Candida* spp. Unconsciousness, prolonged hospitalization, transfusion, mechanical ventilation, use of central venous catheter, enteral feeding via a nasogastric tube, urinary catheter, and receiving carbapenems and glycopeptides were found to be significantly higher in NIs patients. Multivariate logistic regression analysis showed prolonged hospitalization, neutropenia, and use of central venous catheter and carbapenems as the independent risk factors for NIs. In the univariate analysis, unconsciousness, mechanical ventilation, enteral feeding, use of enteral feeding via a nasogastric tube, H_2_ receptor blockers, and port and urinary catheter were significantly associated with mortality. In the multiple logistic regression analysis, only mechanical ventilation was found as an independent predictor of mortality in patients with NIs.

## 1. Introduction

Healthcare-associated infections (HC-AIs) are becoming an increasingly important health issue worldwide. HC-AIs, which are generally observed among patients receiving healthcare in hospital settings, are associated with increased morbidity, mortality, and duration of hospitalization, as well as increased treatment duration and costs. Patients hospitalized in pediatric services and pediatric intensive care units (PICUs) have a higher risk of nosocomial infections [1]. Prolonged hospitalization, invasive interventions, congenital malformations, and total parenteral nutrition are significant factors that increase the risk of nosocomial infections in pediatric patients [2]. Knowing the risk factors that lead to healthcare-associated infections in pediatric patients is important for preventing nosocomial infections and reducing associated mortality. There are limited number of studies investigating the risk factors and mortality of nosocomial infections in pediatric services.

In this study, our aim was to determine the frequency, type, and most common underlying factors of nosocomial infections among patients hospitalized in the Pediatrics Clinics and evaluate the effects of these infections on the duration of hospitalization and the independent risk of nosocomial infections.

## 2. Materials and Methods

The study was performed between September 2011 and April 2014 in patients who were hospitalized, monitored, and treated in the patient services and PICU of the Dicle University Children Hospital, excluding patients at the neonatal service; all patients included in the study were hospitalized for more than 48 hours. A total of 86 patients and 86 control subjects were included in the study. Surveillance of the nosocomial infection was performed actively and prospectively by a physician and a nurse, who checked the patient infections. Patients were visited every day throughout their period of hospitalization, and information regarding the patients was recorded on the patient follow-up chart of the infection control committee. Nosocomial infections were diagnosed according to the Centers for Disease Control and Prevention (CDC) criteria, which were based on the patients' clinical and physical examination findings and laboratory results.

Blood, urine, sputum, cerebrospinal fluid, wound site, and catheter culture samples were obtained from every patient assumed to have developed HAI; endotracheal aspirate samples were also obtained from patients receiving mechanical ventilation. BACTEC Peds Plus/F (BD, Sparks MD)® culture bottles were used for collecting blood samples. The Automatized Phoenix culture system was used to identify the microorganisms and their antibiotic sensitivities. The reports prepared according to the standards of the Clinical and Laboratory Institute, USA, were carefully evaluated. Clinical and laboratory findings were thoroughly evaluated at the time of diagnosis. Laboratory results included positive cultures, which were presumably obtained from sterile sites (blood, CNS-fluid, and pleural and peritoneal fluids), as well as peripheral leukocyte and platelet counts and C-reactive protein (CRP) levels. New infiltrations on radiological images were also examined. The patients' clinical presentations included fever, pulmonary auscultation findings, and hypotension. The clinical, radiological, laboratory, and culture results of HAI patients were recorded on the standard forms of the NNIS-system, and all relevant data were recorded daily onto computers. HAI diagnosis was established according to the criteria set by the CDC.

Peripheral blood culture samples and blood samples obtained via sterile catheters were collected from patients monitored at pediatric services and PICU who were suspected of having bacteremia. These samples were assayed with the BacT/Alert (Biomerieux, France) automated blood culture system; positive-testing samples were subjected to further evaluation. Three to four cm sections from the tip of central venous catheters were seeded semi-quantitatively under sterile conditions according to the Maki method into blood agar and EMB agar. Samples of urine and tracheal aspirate were collected under sterile conditions and seeded into sheep blood agar and EMB plates. The diagnosis of urinary tract infection (UTI) was done by the detection of bacteria in the urine sample drawn from the catheter with at least 100.000 CFU/mL of a single or two different species. A fully automated identification and antibiogram device was used for the bacterial identification and antibiogram.

Patients without nosocomial infections who were hospitalized at the pediatric clinic throughout study period were recruited as controls. Control subjects were specifically selected from patients hospitalized at the same clinic and with similar ages.

Prolonged hospitalization is defined as hospitalization equal or longer than two weeks. Neutropenia is defined as an absolute neutrophil count of less than 500/*μ*L or less than 1000/*μ*L with an anticipated decline to less than 500/*μ*L in the next 48-hour period.

Ethical approval for the study was obtained from the Noninterventional Clinical Research Ethics Committee of Dicle University Medical Faculty.

## 3. Statistical Analysis

Data were analyzed using the SPSS 18.0 (SPSS Inc., Chicago, IL, USA) statistical software. Visual (histogram) and analytical methods (Kolmogorov-Smirnov test) were used to determine whether variables exhibited normal distribution. Variables with normal distribution were expressed as mean plus/minus standard deviation, while not normally distributed data were expressed as median (minimum-maximum) values. Independent groups were compared by Student's *t*-test. Categorical data were compared using the Chi-square test. Correlations were determined using Pearson's or Spearman's correlation analyses. To identify independent factors that may influence disposition, multivariable logistic regression analyses (polytomous responses) were performed to calculate the odds ratio and corresponding 95% confidence intervals. *p* value <0.05 was considered statistically significant.

## 4. Results

The primary outcome of the study was the development of HC-AI. There were 100 episodes of nosocomial infections in 86 patients. Among these cases with hospital infections, 43 were female (50%) and 43 were male (50%). Of the control subjects, 48 were male (55.9%) and 38 were female (44.1%). The median age and interquartile ranges (25th and 75th percentile) of the patients and control group were 32.0 (9.0–58.0) months and 57.5 (33.5–91.0) months, respectively. There were no significant differences in the mean age and gender distribution between the study and the control groups (*p* > 0.05). The median duration of hospital stay and interquartile ranges (25th and 75th percentile) of the patients and control group were 30.0 (20.0–51.0) days and 7.0 (3.0–12.0) days, respectively. Ventilator utilization ratio was 0.58 and central venous catheter utilization ratio was 0.24. The VAP rate was 3.65 per 1000 ventilator days, and the catheter associated bloodstream infection rate was 6.48 per 1000 catheter days. When patients with HC-AI were evaluated with respect to the hospitalized departments, it was determined that 45 (52.3%) were monitored in PICU, 26 (30.2%) were in the pediatric hematology clinic, and 15 (17.5%) were monitored in other clinics. In the control group, 31 (36%) subjects were monitored at the PICU, 18 (21%) were monitored at the pediatric hematology clinic, and 37 (43%) were monitored at other clinics. No significant difference was found in clinical distribution between patients with and without NIs (*p* > 0.05). The most frequent types of infections were bloodstream infections, ventilator-associated pneumonia, catheter associated bloodstream infections, and urinary tract infections (Table 1). The most commonly isolated microorganisms were Coagulase-negative* Staphylococcus* (20%),* Klebsiella *spp. (17.8%),* Acinetobacter* spp. (17.8%), and* Candida* spp. (17.8%). Methicillin-Resistant* S. aureus* (MRSA) was found less than 1% of all NIs isolated microorganisms. And extended spectrum betalactamases (ESBL) ratio was observed with 69 percentage.

Univariate analysis showed that unconsciousness, prolonged hospitalization (2 weeks), transfusion, mechanical ventilation, enteral feeding via a nasogastric tube, use of central venous catheter, urinary catheter, carbapenems, and glycopeptides were significantly associated with nosocomial infections (Table 2). Variables with *p* value <0.05 were selected for the logistic regression model. Nine variables (prolonged hospitalization, neutropenia, transfusion, mechanical ventilation, use of central venous catheter, enteral feeding via a nasogastric tube, carbapenems, aminoglycosides, and glycopeptides) were selected among other similar variables based on clinical judgement. In multiple logistic regression analysis, prolonged hospitalization, neutropenia, use of central venous catheter, and carbapenems were found to be independent risk factors for nosocomial infections among patients with HC-AIs (*R* square = 0.544) (Table 3).

In the univariate analysis, unconsciousness, mechanical ventilation, enteral feeding, use of enteral feeding via a nasogastric tube, H_2_ receptor blockers, and port and urinary catheter were found to be significantly associated with mortality. Variables with *p* value <0.05 were selected for logistic regression model. Due to the small sample size (*n* = 26), we selected three variables (mechanical ventilation, use of H_2_ receptor blockers, and use of urinary catheter) among similar variables based on clinical judgement. In the multiple logistic regression analysis, only mechanical ventilation (OR: 21.7, 95% CI, 2.94–160, *p* = 0.003, *R* square: 0.504) was determined to be an independent predictor of mortality in patients with nosocomial infection (Table 4).

## 5. Discussion

Healthcare-associated infections (HC-AIs) are the most important causes of morbidity and mortality in the pediatric clinic patients. Recently, with the improvement in the likelihood of survival of pediatric patients who have been under risk, HC-AIs are becoming an increasingly important problem in PICUs [3]. In pediatric patients, HC-AIs are a determining factor of morbidity and mortality; therefore, it is important to know which factors are associated with the development of infections in children, a population on which there are only limited studies. Determining the risk factors of HC-AIs may help decrease the incidence of infections and reduce healthcare costs. On the other hand, long-term monitoring, invasive interventions, total parenteral nutrition, and the use of high-spectrum antibiotics are factors that increase the risk of infection among patients who are treated and monitored in pediatric clinics, especially in PICUs and pediatric hematology clinics [4–6]. In parallel with the findings of earlier studies, the frequency of nosocomial infections was highest in our study among PICU patients.

The most frequently observed infections in our study were bloodstream infections, ventilator-associated pneumonia, and urinary tract infections. The ratio of nosocomial infections varies between 7% and 24% depending on the following risk factors: the number of patients being in the care, frequency of invasive interventions, number of skilled health personnel, medical equipment and infrastructure, and the types of medical treatment offered [1]. A systematic review performed by Balaban et al. in Turkey reported results similar to those of our study [2]. Nosocomial infections increase not only morbidity and mortality rates, but also responsible prolongation of hospitalization and increase healthcare costs [7–9]. The outcome of nosocomial infections depends on the causative pathogen; for example, if bloodstream infections are due to Coagulase-negative* Staphylococci*, they are associated with increased mortality. In addition, the sensitivity of the pathogen to the empirical antimicrobial treatment may play an important role [4, 6].

Microorganisms which are responsible for nosocomial infections vary not only from year to year but also between countries [5].* S. aureus* was a frequently identified as causative agent in previous years, while in the following years and today, Gram-negative microorganisms and Coagulase-negative* Staphylococci* are the most frequently identified causative pathogens [10]. In our study, MRSA represented a very low percentage (less than 1%) of causative agents. An earlier study performed by Erdem et al. indicated that the incidence of* S. aureus* infections was declining rapidly in Turkish intensive care units (ICUs), with potential implications on empirical treatment strategies [11].* Acinetobacter* with multiple drug resistance also represent a serious problem recently. This problem is mainly associated with long-term hospitalization, use of inappropriate antibiotics, and failure to follow adequately rules of infection control. In our study, prolonged use of wide-spectrum antibiotics was determined to be a risk factor for the HC-AIs. Similarly in the study of Tekin et al., prolonged use of wide-spectrum antibiotics was determined to be a risk factor for multidrug-resistant* Acinetobacter* [12].* Acinetobacter* infections are associated with increase in mortality rates and prolongation of hospital stay. Therefore, unnecessary antibiotic use and prolonged hospitalization should be avoided, especially in patients who were treated and monitored in PICUs and hematology clinics.

We observed a mortality rate of 30.2% in present study, which is comparatively higher than the ratio reported by another study that identified a mortality rate of 27.5% [2]. We determined that unconsciousness, mechanical ventilation, enteral feeding, use of enteral feeding via a nasogastric tube, H_2_ receptor blockers, and existence of port and urinary catheters are the risk factors that lead to increased mortality rate among pediatric patients. Mechanical ventilation was determined to be an independent predictor of mortality in patients with nosocomial infections. In a study of Hacımustafaoğlu et al., endotracheal intubation, urinary catheter, and male gender were determined to be independent risk factors for mortality [5]. The presence of independent risk factor in our study group and the predominance of ventilator-associated pneumonia were associated with higher mortality rate. Due to the relatively limited number of patients in our study, we suggest that further comprehensive prospective studies need to be conducted in order to better determine the factors affecting mortality.

Determining the variable factors associated with nosocomial infections and taking the necessary measures against them will help reduce morbidity and mortality rates. It is possible to reduce the incidence of HC-AIs through a number of significant strategies and practices. These should include measures such as appropriate hand washing, before and after every contact with patients; periodic training of the health workers; ensuring hygiene in the clinical environment; developing principles on the use of central venous catheters; limiting the use of invasive catheters; and rational use of antibiotics for treatment and prophylaxis of infections. In addition, conducting regular surveillance activities in hospitals and reviewing the associated surveillance data, determining the potential causative infectious agents in ICUs, and detecting resistance of infectious agents to antibiotics will contribute to the management of nosocomial infections [13, 14].

In conclusion, nosocomial infections represent a particularly important issue in pediatric clinics and PICUs. Close monitoring may decrease the rates of healthcare infections and mortality. Measures for controlling infections, such as ensuring compliance to hand hygiene practices, reducing the duration of hospital stay for patients, and preventing improper use of antibiotics, all will contribute to reducing the incidence of nosocomial infections and related mortality.

## Figures and Tables

**Table 1:** Distribution of nosocomial infections according to the infection sites.

Type of nosocomial infection	Number (%)
Bloodstream infection	33 (33)
Ventilator-associated pneumonia	19 (19)
Catheter associated bloodstream infection	14 (14)
Urinary tract infection	13 (13)
Urinary catheter related infection	7 (7)
Shunt infection	3 (3)
Pneumonia	2 (2)
Gastrointestinal infection	2 (2)
Skin and soft tissue infection	2 (2)
Nosocomial meningitis	2 (2)
Surgical site infection	2 (2)
Decubitus ulcer	1 (1)

Total	**100 (100)**

**Table 2:** Comparison of risk factors between infected and uninfected groups.

Risk factors	Uninfected patients (*n* = 86)	Infected patients (*n* = 86)	*p*
Sex %			
Female	55.9	50.0	0.637
Male	44.1	50.0	
Age (month)^*∗*^	32.0 (9.0–58.0)	57.5 (33.5–91.0)	0.286
Intensive care stay %	36.0	52.3	0.061
Length of hospital stay (days)^*∗*^	30.0 (20.0–51.0)	7.0 (3.0–12.0)	**<0.001**
Unconsciousness %	16.2	46.5	**<0.001**
Prolonged hospitalization %	12.8	67.4	**<0.001**
Use of central venous catheter %	2.3	24.4	**<0.001**
Enteral feeding via a nasogastric tube %	23.2	54.6	**<0.001**
Transfusion %	17.4	53.4	**<0.001**
Parenteral nutrition %	6.9	17.4	**0.036**
Use of H_2_ receptor blockers %	25.5	43.0	**0.016**
Mechanical ventilation %	13.9	45.3	**<0.001**
Use of urinary catheter %	4.6	43.0	**<0.001**
Neutropenia %	6.9	23.2	**0.003**
Use of port %	4.6	13.9	**0.036**
Use of third-generation cephalosporins %	24.4	38.3	**0.049**
Use of carbapenems %	11.6	55.8	**<0.001**
Use of steroid %	8.1	6.9	0.773
Use of aminoglycosides %	3.4	24.4	**0.001**
Use of glycopeptides %	4.6	38.3	**<0.001**

^*∗*^Median and interquartile ranges (25th and 75th percentile).

**Table 3:** Multivariate logistic regression analysis in prediction of independent risk factors for hospital infections.

	Unadjusted		Adjusted	
	OR (95% CI)	*p*	OR (95% CI)	*p*
Prolonged hospitalization	14.1 (6.49–30.7)	<0.001	4.64 (1.75–12.3)	**0.002**
Neutropenia	4.04 (1.53–10.6)	0.003	5.31 (1.21–23.3)	**0.027**
Use of central venous catheter	13.5 (3.07–59.9)	<0.001	6.19 (1.05–36.3)	**0.043**
Transfusion	5.44 (2.70–10.9)	<0.001	0.44 (0.13–1.48)	0.188
Enteral feeding via a nasogastric tube	3.97 (2.06–7.66)	<0.001	3.31 (0.94–11.5)	0.061
Mechanical ventilation	5.11 (2.43–10.7)	<0.001	1.13 (0.29–4.34)	0.857
Use of carbapenems	9.47 (4.32–20.7)	<0.001	3.09 (1.09–8.76)	**0.034**
Use of glycopeptides	12.7 (4.27–38.1)	<0.001	3.18 (0.84–11.9)	0.087
Use of aminoglycosides	5.89 (1.90–18.2)	0.001	2.23 (0.55–9.00)	0.258

*R square = 0.544*.

**Table 4:** Multivariate logistic regression analysis in prediction of independent risk factors for mortality.

	Unadjusted		Adjusted	
	OR (95% CI)	*p*	OR (95% CI)	*p*
Unconsciousness	0.07 (0.02–0.25)	<0.001		
Mechanical ventilation	0.02 (0.06–0.13)	<0.001	21.7 (2.94–160)	** 0.003**
Use of enteral feeding via a nasogastric tube	0.05 (0.01–0.24)	<0.001	2.89 (0.34–24.5)	0.329
Use of H_2_ receptor blockers	0.21 (0.07–0.55)	<0.001	1.34 (0.30–5.92)	0.691
Enteral feeding	0.03 (0.04–0.25)	<0.001		
Use of port	0.64 (0.54–0.76)	<0.014		
Use of urinary catheter	0.26 (0.10–0.69)	<0.006		
